# Mapping of a hybrid insulin peptide in the inflamed islet β-cells from NOD mice

**DOI:** 10.3389/fimmu.2024.1348131

**Published:** 2024-02-22

**Authors:** Janet M. Wenzlau, Orion J. Peterson, Anthony N. Vomund, James E. DiLisio, Anita Hohenstein, Kathryn Haskins, Xiaoxiao Wan

**Affiliations:** ^1^Department of Immunology and Microbiology, University of Colorado School of Medicine, Aurora, CO, United States; ^2^Division of Immunobiology, Department of Pathology and Immunology, Washington University School of Medicine, St. Louis, MO, United States; ^3^Bursky Center for Human Immunology and Immunotherapy Programs, Washington University School of Medicine, St. Louis, MO, United States

**Keywords:** autoimmune diabetes, type 1 diabetes, hybrid insulin peptide, antigen, post-translational modification, NOD, islets, β-cells

## Abstract

There is accumulating evidence that pathogenic T cells in T1D recognize epitopes formed by post-translational modifications of β-cell antigens, including hybrid insulin peptides (HIPs). The ligands for several CD4 T-cell clones derived from the NOD mouse are HIPs composed of a fragment of proinsulin joined to peptides from endogenous β-cell granule proteins. The diabetogenic T-cell clone BDC-6.9 reacts to a fragment of C-peptide fused to a cleavage product of pro-islet amyloid polypeptide (6.9HIP). In this study, we used a monoclonal antibody (MAb) to the 6.9HIP to determine when and where HIP antigens are present in NOD islets during disease progression and with which immune cells they associate. Immunogold labeling of the 6.9HIP MAb and organelle-specific markers for electron microscopy were employed to map the subcellular compartment(s) in which the HIP is localized within β-cells. While the insulin B9-23 peptide was present in nearly all islets, the 6.9HIP MAb stained infiltrated islets only in NOD mice at advanced stages of T1D development. Islets co-stained with the 6.9HIP MAb and antibodies to mark insulin, macrophages, and dendritic cells indicate that 6.9HIP co-localizes within insulin-positive β-cells as well as intra-islet antigen-presenting cells (APCs). In electron micrographs, the 6.9HIP co-localized with granule structures containing insulin alone or both insulin and LAMP1 within β-cells. Exposing NOD islets to the endoplasmic reticulum (ER) stress inducer tunicamycin significantly increased levels of 6.9HIP in subcellular fractions containing crinosomes and dense-core granules (DCGs). This work demonstrates that the 6.9HIP can be visualized in the infiltrated islets and suggests that intra-islet APCs may acquire and present HIP antigens within islets.

## Introduction

A broad variety of self-antigens have been implicated in autoimmune diabetes, but only a subset of these is likely to be involved in disease initiation, with subsequent epitope spreading to other antigens. In the NOD mouse, substantial evidence supports proinsulin as the predominant instigating antigen (1–3). Efforts to define which proinsulin-derived epitopes are most pertinent to disease etiology have included a wide variety of approaches, including genetics, biochemistry, confocal and electron microscopy (EM), and targeted mass spectrometry. These studies have revealed different forms of insulin-derived epitopes, including native epitopes of insulin B-chain and C-peptide, as well as a class of modified antigens, hybrid insulin peptides (HIPs) resulting from peptide fusion, all of which contribute to the activation of pathogenic CD4 T cells.

Insulin secretory granules are considered a repository of antigenic targets involved in autoimmune diabetes (4). However, the subcellular localization of immunogenic insulin-derived epitopes in β-cell granules remains largely unexplored. The primary secretory granule in β-cells is the insulin dense-core granule (DCG), containing a crystal core of insulin. The DCG has diverse biological functions, such as proinsulin processing and insulin secretion. Maintaining insulin homeostasis in β-cells is achieved by fusing excessive DCGs with lysosomes for catabolic reduction. This crinophagic pathway generates a minor set of β-cell granules, termed crinophagic bodies or crinosomes, which possess lysosomal activities to catabolize secretory proteins in DCGs (5–7).

The subcellular distribution of insulin-derived antigens that elicit the most robust T-cell responses within β-cells has been probed using differential centrifugation to fractionate DCGs and crinosomes with subsequent mass spectrometry to identify the peptides therein (8). The DCG fraction obtained at a high speed (25,000 × *g*) primarily contained intact insulin, long B-chain peptides, and some C-peptides, whereas the granule fraction obtained at a lower speed (5,000 × *g*), representing crinosomes, was enriched for shorter insulin peptides, including shorter B-chain peptides associated with the predominant epitope B9-23 and diverse C-peptide sequences. Further investigation of crinosome-derived peptides, initially by manual investigation of unassigned spectra, revealed a few HIP sequences (8), suggesting the presence of HIPs in crinosomes. These results were later confirmed in a subsequent study (9) by searching the crinosome peptidome against an *in silico* HIP database (10). This approach also identified HIPs in the secretome (peptide contents exocytosed from β-cells upon glucose stimulation) (9), raising the possibility that HIPs are also present in DCGs.

HIPs are the product of a unique post-translational modification whereby insulin peptide fragments are fused to sequences from endogenous proteolytically processed β-cell secretory granule proteins. One such HIP, the 6.9HIP, is composed of a fragment of C-peptide fused to IAPP2 (DLQTLAL/NAARD) and is the antigenic ligand of diabetogenic CD4+ T-cell clone BDC-6.9 from the NOD mouse (11). T cells reactive to the human counterpart of this HIP and others have been identified in PBMCs from T1D patients (12) and distinct HIP-reactive T cells have been cultured from the islets of deceased T1D organ donors (13–16). Importantly, a nearly identical sequence to the 6.9HIP (LQTLAL/NAARD) was identified in the MHC-II (I-Ag7) peptidomes of both pancreatic islets and draining lymph nodes in NOD mice (9), indicating that 6.9HIP is a *bona fide* neoepitope that is presented *in vivo* by MHC-II.

Mapping the intracellular site of HIP formation is challenging, yet essential for putative intervention strategies. Crinosomes and DCGs are both favorable environments for HIP formation due to high concentrations of the peptide components of HIPs and enzymes that function at low pH. Proposed mechanisms for HIP formation via enzymes cathepsin D and cathepsin L have been reported from *in vitro* studies (17, 18). A monoclonal antibody (MAb) specific for the insulin B-chain sequences B9-23, but not native insulin, has been employed to localize the chief epitope B9-23 to crinosomes (8). The B9-23 MAb co-localizes with the lysosomal marker LAMP1 in vesicles distinct from DCGs, providing a punctate staining pattern with immunofluorescent microscopy. Diabetogenic effector T cells reactive to the B12-20 epitope are responsive to the contents of these crinosomes, suggesting that other critical antigens could be present in the same organelles (8, 19). In the current study, we developed a MAb to the C-peptide/IAPP2 (6.9) HIP, previously identified by mass spectrometry, to determine whether such rare post-translationally modified peptides can be detected through microscopic analysis of antibody staining. The primary goals were to establish in which cellular compartment the 6.9HIPs are localized and with what type of antigen-presenting cells (APCs) they may be associated.

## Materials and methods

### Mice

NOD/ShiLtJ (NOD) mice were originally obtained from The Jackson Laboratory. All mice were bred, maintained, and used in experiments in our pathogen-free animal facility in accordance with the guidelines of the Division of Comparative Medicine at Washington University School of Medicine (Association for Assessment and Accreditation of Laboratory Animal Care accreditation no. A3381-01). NOD.IAPP^−/−^ mice were previously bred in the Haskins mouse colony by backcrossing C57BL6.IAPP^−/−^ mice (20) onto the NOD background (21).

### Generation and characterization of the 6.9HIP monoclonal antibody

BALB/c mice were immunized three times, 2 weeks apart with 100 µg/each of the NOD 6.9HIP (SLDQLALNAARDPN) conjugated to KLH and combined 1:1 with CFA, IFA, and IFA, respectively. Sera were monitored for MAb titer with enzyme-linked immunosorbent assays (ELISAs) and radio binding assays [as in (22)] using a recombinant probe expressing the NOD 6.9HIP. A MAb targeting the NOD 6.9HIP sequence was developed as previously described (23). Specificity of the 6.9HIP MAb was determined using Western blots and in-house ELISAs, using anti-mouse HRP 1:10,000 (Pierce) and quantitation with o-phenylenediamine dihydrochloride substrate at 490 nm as per vendor recommendations (Sigma).

### Recombinant proteins and peptides

Mouse HIPs were cloned in-frame with fusion partner NUS in vector pET43a (Novagen) for expression in bacteria. Induced proteins were purified on Ni-Agarose (Qiagen) according to the manufacturer’s instructions. 6.9HIP and control peptides (>95% purity, Genscript or Peptide 2.0) were resuspended at a concentration of 10 mg/mL according to solubility recommendations.

### Direct binding ELISA

Ninety-six-well ELISA plates were coated with 6.9HIP peptide (LQTLAL/NAARD, 2 μM) in carbonate buffer overnight at 4°C. Plates were washed and subsequently blocked with DMEM/5% fetal bovine serum (FBS). The 6.9HIP MAb was serially diluted and directly added to the coated plate. Horseradish peroxide (HRP)-conjugated goat anti-mouse IgG (1:10,000) antibody was then added for 2 h at 4°C, the responses were measured using the OptEIA TMB Substrate (BD), and the data [optical density (OD) at 450 nm] were collected using an iMark Microplate Reader (Bio-Rad Laboratories).

### Competitive ELISA

ELISA plates were coated with 6.9HIP peptide (2 μM) in carbonate buffer overnight at 4°C. Plates were washed and subsequently blocked with DMEM/5% FBS. Soluble competitive inhibitors (synthetic peptides) were pre-incubated with the 6.9HIP MAb (50 ng/mL) for 30 min and the mixture was incubated with the plate-bound antigens for 2 h at 37°C. In the absence of soluble competitive inhibitors, the concentration of the MAb resulted in approximately 50% binding to the plate-bound antigen. HRP-conjugated goat anti-mouse IgG (1:10,000) antibody was then added for 2 h at 4°C, the responses were quantified using the OptEIA TMB Substrate (BD), and the data (OD at 450 nm) were collected using an iMark Microplate Reader (Bio-Rad Laboratories).

### Islet isolation

Mouse peritoneal cavities were opened to clamp the common bile duct leading to the duodenum. Type XI collagenase (0.4 mg/mL in serum-free DMEM; Sigma-Aldrich) digestion buffer was injected through the bile duct to inflate the pancreas, which was removed and incubated in digestion buffer at 37°C (12 min) and then shaken vigorously for 90 s. Pancreata were washed with serum-free (SF) DMEM (3×) through a stainless-steel strainer. The flow-through, containing islets, was then filtered through a 70-µm cell strainer. Retained islets were then flushed into a Petri dish for hand-picking. The NOD.IAPP^−/−^ islets were prepared similarly by infusion of pancreata with CIzyme RI (VitaCyte) through the bile duct followed by gravity filtration washes (as above). Islets were enriched with gradient centrifugation using Lympholyte 1.1 (Cedarlane Laboratories) where they concentrate at the interface and were hand-dissected. Pure hand-picked islets were dispersed using Cell Dissociation Solution Non-Enzymatic (Sigma-Aldrich) for 10 min at 37°C.

### Confocal microscopy

Intact islets were fixed in 4% formaldehyde, permeabilized, and blocked in 0.2% saponin/3% bovine serum albumin (BSA). Primary 6.9HIP or AIP antibody (2 µg/mL) was used to stain islets overnight in 0.2% saponin/3% BSA. Anti-mouse-AF594 secondary antibody (4 µg/mL) was permitted to bind islets for 1 h in saponin/BSA. Islets were washed with saponin/BSA and mounted on slides with prolong diamond antifade mounting media (Invitrogen). Islets were blocked with saponin/BSA and then stained with antibodies to cell surface markers (CD11c-488 or CD4-BV480, SIRPα-AF488, and F4/80-AF594) at 2 µg/mL for 1 h followed by washing with saponin/BSA for imaging. For staining with conjugated 6.9HIP MAb, islets were fixed in 4% formaldehyde, permeabilized and blocked with saponin/BSA, and stained with 6.9HIP-AF647 at 0.033 µg/mL overnight in saponin/BSA. Islets were washed and mounted as above for imaging using an inverted Leica SP8 confocal scanning microscope with Leica’s LAS X software. The microscope was equipped with a 63× 1.40 numerical aperture (NA) oil-immersion objective and a white light laser. Optical sections were taken every 0.75 µm. Images shown were taken with Imaris 9.0 software.

### Immunogold transmission electron microscopy

For immunolocalization at the ultrastructural level, islets were fixed in 4% paraformaldehyde/0.05% glutaraldehyde (Polysciences) in 100 mM PIPES/0.5 mM MgCl_2_, pH 7.2, for 1 h at 4°C. Samples were then embedded in 10% gelatin and infiltrated overnight with 2.3 M sucrose/20% polyvinyl pyrrolidone in PIPES/MgCl_2_ at 4°C. Samples were trimmed, frozen in liquid nitrogen, and sectioned with a Leica Ultracut UCT7 cryo-ultramicrotome (Leica Microsystems). Ultrathin sections of 50 nm were blocked with 5% FBS/5% normal goat serum for 30 min and subsequently incubated with indicated primary antibodies for 1 h at room temperature. Following washes in block buffer, sections were then incubated with goat anti-mouse IgG conjugated to 18-nm colloidal gold, anti-rat IgG conjugated to 12-nm colloidal gold, and goat anti-rabbit IgG antibody conjugated to 6-nm colloidal gold (Jackson ImmunoResearch Laboratories) for 1 h. Sections were stained with 0.3% uranyl acetate/2% methyl cellulose and viewed on a JEOL 1200 EX transmission electron microscope (JEOL) equipped with an AMT 8-megapixel digital camera and AMT Image Capture Engine V602 software (Advanced Microscopy Techniques). All labeling experiments were conducted in parallel with controls omitting the primary antibody.

### Generation and screen of T-cell hybridomas

T-cell hybridomas were generated by fusion of activated lymph node cells elicited by 6.9HIP peptide immunization with BW5147 fusion partner following standard protocols. The T-cell hybridoma clones were screened for reactivity with the 6.9HIP peptide using the C3.g7 APC line in antigen assays. IL-2 production was assessed via IL-2 ELISA.

### *In vitro* ER stress induction and inhibition

Whole islets were isolated from NOD mice as described and dispersed for 3 min at 37°C using non-enzymatic cell dissociation buffer (Millipore Sigma). Cells were collected by centrifugation, washed with PBS, and counted. Cells were resuspended in DMEM/10% FBS low-glucose media (1 g/L glucose, Gibco, D10F-LG) and split into 1.0 mL aliquots in microfuge tubes for treatment overnight at 37°C in D10F-LG or D10F-LG containing 100 µM tauroursodeoxycholic acid (TUDCA; Millipore Sigma). Tunicamycin (Millipore Sigma) was added at 1 µg/mL to appropriate samples and was incubated for 2 h at 37°C, after which cells were collected, washed, and counted for antigen assays.

### Granule isolation and presentation assay

Islet cells were lysed in 1.0 mL of phosphate-buffered saline (PBS) by passage through a cell homogenizer (Isobiotec) 5×. The cell lysate was spun 2× for 10 min at 500 × *g* at 4°C to remove cell debris. The supernatants were then sequentially spun 2× for 10 min at 5,000 × *g* at 4°C, and the cell pellets were resuspended in media and pooled as the crinosome fraction. The supernatant was spun for 30 min at 25,000 × *g* at 4°C, and the pellet was resuspended in media as the DCG fraction. For presentation, the isolated crinosomes (5,000 × *g* pellet) or DCGs (25,000 × *g* pellet) were offered to the C3.g7 APC line (B cell lymphoma expressing I-Ag^7^; 5 × 10^4^/well) in a 96-well culture plate. After a 2 h incubation, CD4+ T-cell hybridomas (5 × 10^4^/well) were added.

### Antigen presentation assay

APCs were cultured overnight with T-cell hybridomas (6.9-11, 6.9-51, and 6.9-61) recognizing the 6.9HIP. The T-cell responses were tested for IL-2 production measured by IL-2 ELISA (Bectin Dickinson) coupled with streptavidin-poly HRP (Thermo Fisher). The responses were quantified using the OptEIA TMB Substrate (Bectin Dickinson), and the data (OD at 450 nm) were collected using an iMark Microplate Reader (Bio-Rad Laboratories).

## Results

### Characterization of the 6.9HIP monoclonal antibody

**Figure 1:**
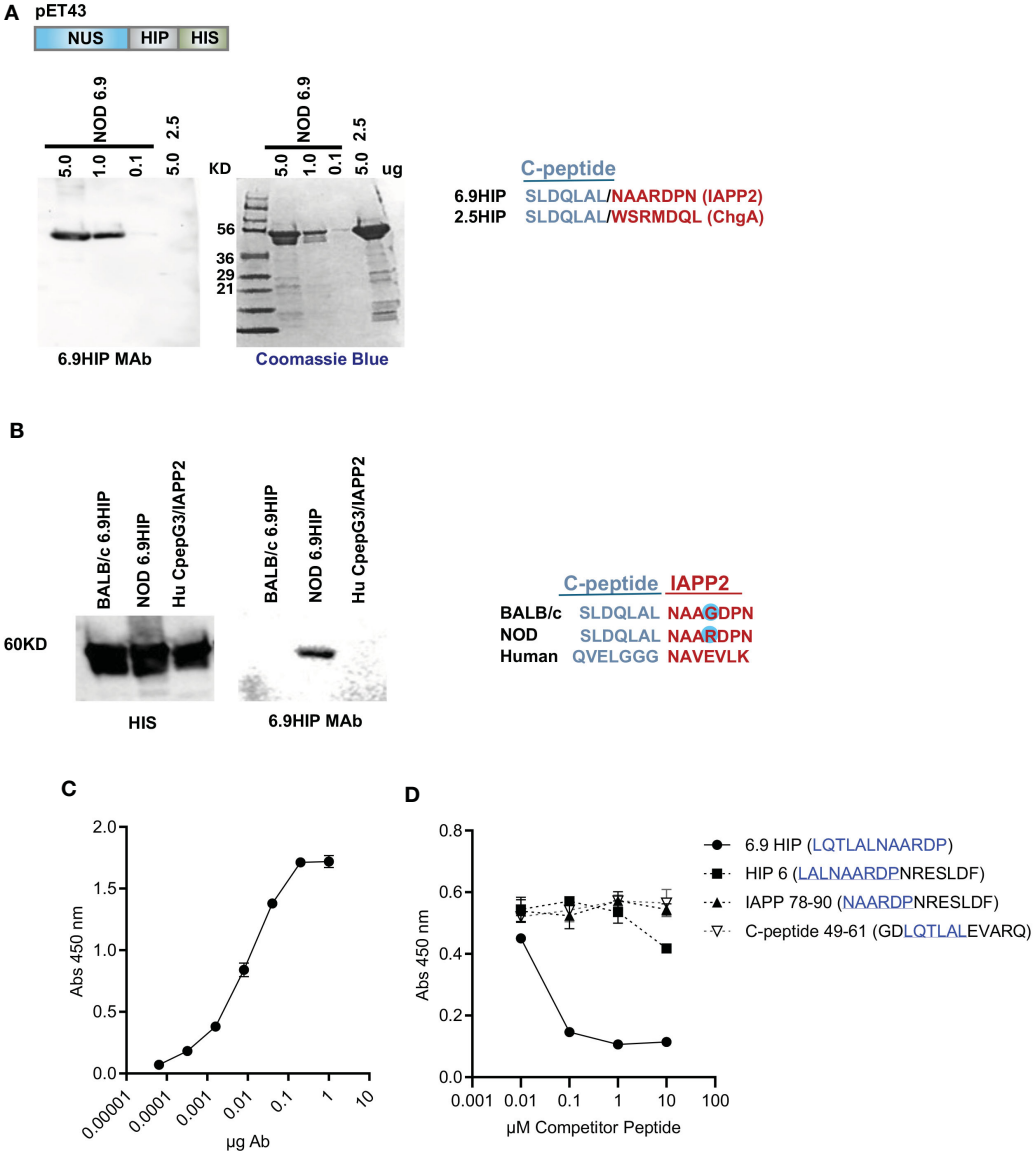
Characterization of 6.9HIP MAb. **(A)** Western blot (with 6.9MAb) and Coomassie blue-stained gel with titration of recombinant NUS-6.9HIP (5.0–0.1 mg) and NUS-2.5HIP (5 mg) fusion proteins. HIP sequences are aligned on the right. **(B)** Western blots with recombinant BALB/c 6.9HIP, NOD 6.9HIP, and HuC-pepG3/IAPP2 HIP proteins probed with HIS and 6.9HIP primary antibodies and goat anti-mouse-HRP secondary antibodies. HIP sequences are aligned below. **(C)** ELISA showing the binding of the 6.9HIPMAb to plate-bound 6.9HIP peptide. **(D)** Competitive ELISA showing the inhibition of the 6.9MAb:6.9HIP binding by indicated competitor peptides. The peptide sequences related to the 6.9HIP are underlined.

Within the islets, HIPs are present at very low concentrations (13). To evaluate the specificity of the putative 6.9HIP (SLDQLAL/NAARDPN) MAb, we generated a set of NUS/HIP recombinant fusion proteins (expressed in the vector pET43) including the NOD 6.9HIP, the BALB/c 6.9HIP, the 2.5HIP, and a human C-peptide/IAPP2 HIP and queried the ability of 6.9HIP MAb to bind to each using Western analysis (**Figures 1A, B**). Results showed that the 6.9MAb bound specifically to the NOD 6.9HIP fusion protein and not to the 2.5HIP fusion protein, which shares the identical seven-amino-acid C-peptide region (**Figures 1A, B**, blue). In a similar experiment, fusion proteins 6.9HIP BALB/c and 6.9HIP NOD, which differ by one amino acid within the IAPP2 segment (R>G), and a human peptide (Hu CpepG3/IAPP2) with significant differences in both C-peptide and IAPP2 regions were all detected by blotting with an anti-HIS antibody, indicating approximately equal protein loading (**Figure 1B**, left) but only the 6.9HIP NOD peptide was detected by the 6.9HIP MAb (**Figure 1B**, right).

ELISA confirmed that reactivity of 6.9HIP MAb against plate-bound 6.9HIP peptide (LQTLAL/NAARDP) was strictly dose-dependent (**Figure 1C**). To test the level of specificity, a competitive ELISA was performed, in which the 6.9HIP MAb was preincubated with different concentrations of one of four competitor peptides, including the synthetic 6.9HIP and three other peptides containing a portion of the 6.9HIP (see **Figure 1D** for details). The 6.9HIP MAb:peptide mixtures were then incubated with the plate-bound 6.9HIP peptide, and the binding was assayed by ELISA. As shown in **Figure 1D**, only the 6.9HIP MAb:6.9HIP peptide combination blocked the binding of the 6.9HIP MAb to plate-bound 6.9HIP, indicating the high specificity of the MAb for 6.9HIP.

### Visualization of 6.9HIP in pancreatic islets

**Figure 2:**
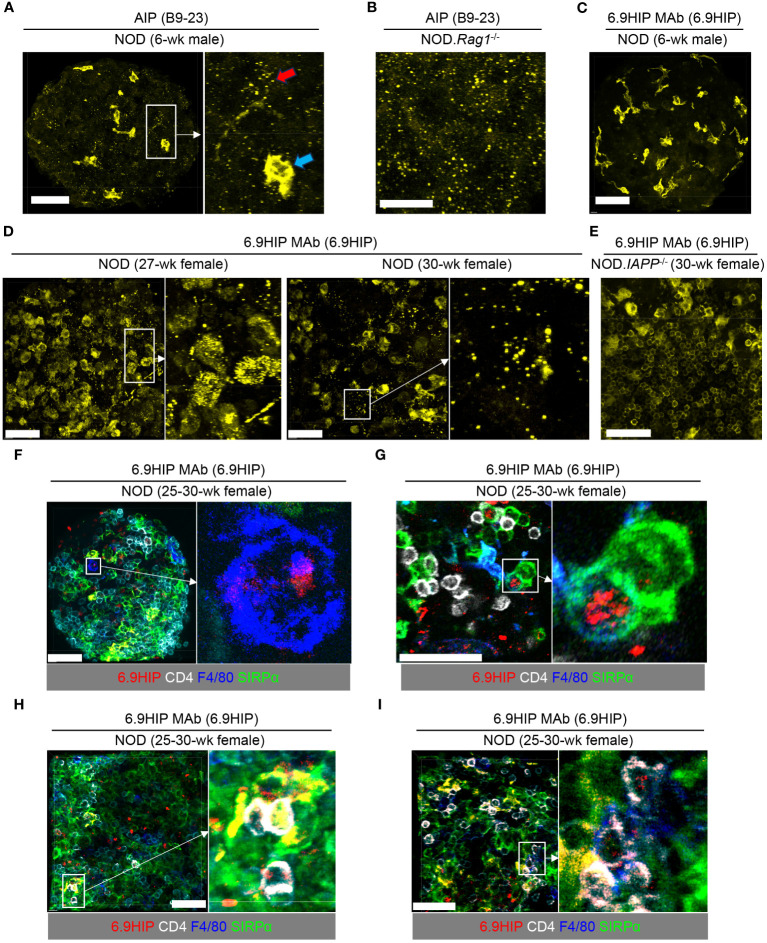
Visualization of 6.9HIP in inflamed islets by confocal microscopy. **(A–E)** Intact islets isolated from NOD mice of indicated sex and age were stained with unconjugated AIP **(A, B)** or 6.9HIP MAbs **(C–E)** followed by a secondary fluorochrome-conjugated anti-mouse IgG F(ab)2 antibody. **(A)** A representative islet (left) from 6-week-old male NOD mice stained with the AIP antibody. A selected region (rectangle) is enlarged in inset (right), showing punctate peptide (B9-23) staining (red arrow), and staining of intra-islet phagocytes by secondary antibody (blue arrow). **(B)** A representative islet from NOD.*Rag1*^-/-^ mice stained with the AIP antibody. **(C)** A representative islet from 6-week-old male NOD mice stained with the 6.9HIP MAb. **(D)** Two representative islets from 27- and 30-week-old female NOD mice stained with the 6.9HIP MAb. Enlarged images on the right show the punctate staining of the 6.9HIP distributed within the islets. **(E)** A representative islet from 30-week-old female NOD.*IAPP*^-/-^ mice stained with the 6.9HIP MAb. **(F–I)** Intact islets isolated from 25- to 30-week-old female NOD mice were stained with fluorochrome-conjugated antibodies to 6.9HIP (red), CD4 (white), F4/80 (blue), and Sirpα (green). **(F, G)** Representative islets showing the co-localization of 6.9HIP with F4/80+ macrophages **(F)** or Sirpα+ cDC2 **(G)**. **(H, I)** Representative islets showing the presence of 6.9HIP when CD4+ T cells interact with Sirpα+ cDC2 **(H)** or F4/80+ macrophages **(I)**. White scale bars in all images are 50 µm.

MAb AIP, specific for the insulin B9-23 peptide, has previously been used to identify the location of the B9-23 peptide in pancreatic islets by confocal imaging (8). We performed intracellular staining with unconjugated AIP MAb in non-sectioned, intact islets obtained from 6-week-old male NOD or NOD.*Rag1*^-/-^ mice. After extensive washing, the signal was visualized by a fluorochrome-conjugated anti-mouse IgG F(ab′)2 secondary antibody. In young (6 weeks) NOD (**Figure 2A**) and NOD.*Rag1*^-/-^ (**Figure 2B**) mice, we observed strong signals from the AIP antibody, which manifested a speckled pattern of staining, indicating the presence of the B9-23 peptide under conditions of low islet inflammation. It should be noted that the secondary anti-mouse IgG F(ab′)2 antibody also recognized endogenous IgG deposited onto islet-resident macrophages known to express Fcγ receptors at a high level (24, 25). This macrophage staining (**Figure 2A**, blue arrow) was distinguishable from the peptide staining (speckled pattern; **Figure 2A** red arrow) and was only observed in islets from WT NOD (**Figure 2A**) but not NOD.*Rag1*^-/-^ mice that lack endogenous IgG (**Figure 2B**).

We then employed the 6.9MAb to visualize the 6.9HIP in islets. We initially used the same staining protocol as described above. To ask whether 6.9HIP could be detected when islet infiltration is minimal, we examined islets isolated from 6-week-old male WT NOD mice. In contrast to results seen in **Figures 2A, B** for detection of B9-23, we did not observe detectable peptide staining in these islets with the 6.9HIP MAb (**Figure 2C**), suggesting the minimal presence of the 6.9HIP in young mice. Considering that 6.9HIP-specific CD4+ T cells have been shown to accumulate in pancreatic islets in NOD mice at advanced stages of diabetes (26), we hypothesized that levels of 6.9HIP might increase in islets from aged NOD mice. To test this, we stained islets from 25- to 30-week-old non-diabetic female NOD mice with the 6.9HIP MAb. In contrast to results seen in young mice, we found a prominent presence of 6.9HIP in infiltrated islets at both 25- and 30-week time points (**Figure 2D**). Like B9-23, the 6.9HIP also exhibited a punctate staining profile within the islets (**Figure 2D**). These results suggest that autoinflammatory responses in aged NOD mice can promote the formation of the 6.9HIP in pancreatic islets.

To further confirm the specificity of the 6.9HIP staining, we examined islets isolated from age/sex-matched NOD mice lacking *IAPP* (NOD.*IAPP*^-/-^), in which 6.9HIP should be absent. Although islets from NOD.*IAPP*^-/-^ mice also displayed background staining of intra-islet phagocytes, no detectable punctate staining of 6.9HIP was observed (**Figure 2E**), confirming the specificity of the 6.9HIP observed in WT NOD mice. To ask if 6.9HIP could be taken up by intra-islet APCs for antigen presentation to T cells, we used fluorochrome-conjugated 6.9HIP MAb to stain infiltrated islets from 25- to 30-week-old non-diabetic female NOD mice, in conjunction with antibody markers that identify CD4+ T cells (anti-CD4), type 2 conventional dendritic cells (cDC2 and anti-SIRPa), and islet macrophages (anti-F4/80). We observed co-localization of 6.9HIP with islet macrophages (F4/80+, **Figure 2F**) and cDC2 dendritic cells (SIRPα+, **Figure 2G**) in different islets. Such localization was also noted when islet-infiltrating CD4+ T cells were in close contact with cDC2 dendritic cells (**Figure 2H**) and islet macrophages (**Figure 2I**). We examined a total of 150 islet macrophages and 321 cDC2. Among these, 26 macrophages (17.3%) and 47 cDC2 (14.6%) showed co-localization with 6.9HIP, supporting that 6.9HIP is a low-abundance antigen. Additionally, we did not observe detectable 6.9HIP signals in islets of 27-week-old female NOD.*Rag1*^-/-^ mice (**Supplementary Figure 1A**), whereas the presence of B9-23 was evident (**Supplementary Figure 1B**). These results further confirm the association of 6.9HIP with islet inflammation. Overall, our data support the hypothesis that the 6.9HIP can be presented by different intra-islet APCs to CD4+ T cells in inflamed islets.

### Localization of 6.9HIP in β-cell granules

Visualization of 6.9HIP in pancreatic islets by confocal microscopy prompted us to further analyze its localization in β-cells at the ultrastructural level. To achieve this, we employed immunogold EM with the 6.9HIP MAb. This method utilizes secondary antibodies conjugated with colloidal gold of different sizes, enabling the visualization of subcellular locations of specific markers (identified by primary antibodies) by EM. Specifically, 6.9HIP was visualized by staining with the 6.9HIP MAb, followed by the addition of a secondary anti-mouse IgG antibody conjugated to 18-nm gold. To test the hypothesis that 6.9HIP might be localized in β-cell granules, we also included a rabbit anti-insulin antibody paired with secondary anti-rabbit IgG (6-nm) and a rat anti-LAMP1 antibody paired with secondary anti-rat IgG (12-nm). These combinations allowed us to identify granule structures associated with the DCGs, lysosomes, and crinosomes.

Given that the success of immunogold EM depends highly on the antibody clone used, we sought to determine the specificity of the 6.9HIP MAb using infiltrated islets from non-diabetic female NOD (30-week-old) mice. We used a rigorous approach in which the 6.9HIP MAb was used as the only primary antibody but was followed by the addition of all three secondary antibodies: anti-mouse IgG (18-nm), anti-rat IgG (12-nm), and anti-rabbit IgG (6-nm). The majority of the observed spots were of the 18-nm size, indicating the binding of the 6.9HIP MAb specifically with the anti-mouse IgG (**Supplementary Figure 2A**). Spots with a 12- or 6-nm size were only occasionally observed (data not shown). Using the same approach, we tested the primary anti-insulin (**Supplementary Figure 2B**) or anti-LAMP1 (**Supplementary Figure 2C**) antibody in islets isolated from the same mice. Both primary antibodies showed specific interactions with their respective secondary antibodies with a rare background. Overall, these results validated the high specificity of the 6.9HIP MAb and confirmed its suitability for use in immunogold EM.

**Figure 3:**
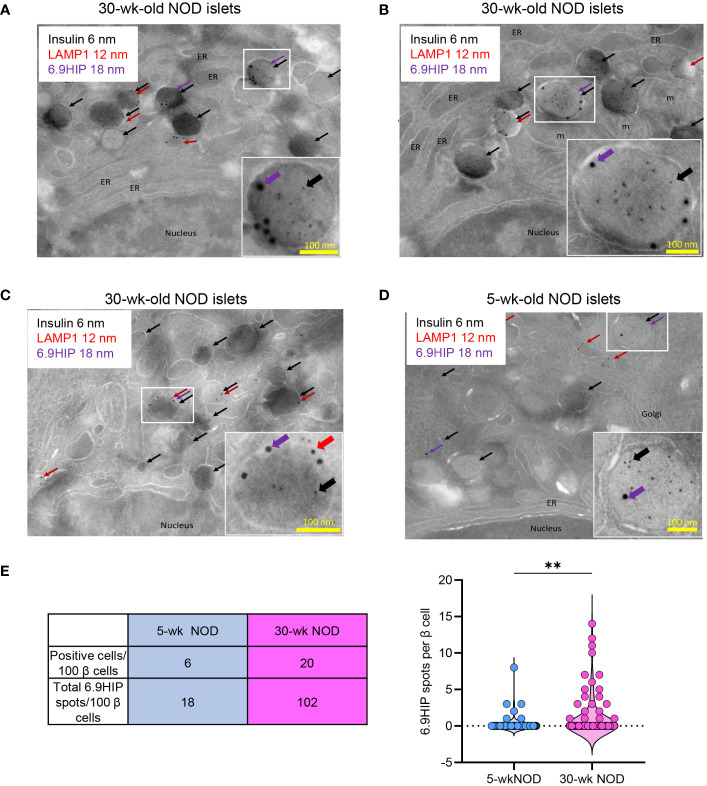
Immunogold electron microscopy reveals that 6.9HIP is localized in two sets of β-cell granules. Islets from 30-week-old **(A–C)** or 5-week-old **(D)** NOD mice were triple labeled with primary rabbit anti-insulin, rat anti-LAMP1, and mouse anti-6.9HIP antibodies, followed by staining with secondary antibodies conjugated to colloidal gold, including anti-rabbit 6-nm (insulin, black), anti-rat 12-nm (LAMP1, red), and anti-mouse 18-nm (6.9HIP, purple). Each image in **(A–D)** shows representative β-cells. The selected regions are illustrated in the enlarged inserts depicting granules containing 6.9HIP in the presence of insulin alone or insulin and LAMP1. ER = endoplasmic reticulum, m = mitochondria. **(E)** The table summarizes the numbers of β-cells containing at least one 6.9HIP spot and the total numbers of 6.9HIP spots quantified from 100 β-cells from either 30- or 5-week-old NOD mice. The violin plot shows the number of 6.9HIP spots from 100 individual β-cells from each condition. Data summarize results from two independent experiments. Each experiment used 200–300 islets isolated from two 5-week-old or five 30-week-old NOD mice. **p < 0.01; Mann–Whitney test.

We then performed triple labeling using all three primary antibodies to assess the localization of the 6.9HIP in different β-cell granules. Within the same β-cell, we identified granule compartments containing insulin alone (DCGs), LAMP1 alone (lysosomes), or both insulin and LAMP1 (crinosomes) (**Figures 3A–D**). In β-cells from non-diabetic female NOD mice (30 weeks old), we observed granules co-labeled with insulin and 6.9HIP, indicating the localization of 6.9HIP within DCGs (**Figures 3A, B**). We also found granules that were simultaneously labeled with insulin, LAMP1, and 6.9HIP, signifying the presence of 6.9HIP within crinosomes (**Figure 3C**). None of the LAMP1 single-positive granules contained 6.9HIP, suggesting minimal presence of 6.9HIP in regular lysosomes. Notably, nearly all the identified spots with different sizes were localized within vesicular structures, with minimal staining in other organelles such as nucleus, mitochondria, or endoplasmic reticulum (ER) (**Figures 3A–D**). These results revealed that 6.9HIPs are specifically localized in both DCGs and crinosomes in β-cells.

In line with our confocal imaging results, the presence of 6.9HIP was less frequent in β-cells from 5-week-old female NOD mice (see **Figure 3D** showing 6.9HIP in DCGs). We examined 100 β-cells for the presence of 6.9HIP in 5- or 30-week-old female NOD mice. Of the 100 β-cells examined, only 6 contained at least one 6.9HIP spot in 5-week-old mice, as compared to 20 β-cells in 30-week-old mice (**Figure 3E**). Furthermore, the number of the 6.9HIP spots across all 100 β-cells was significantly higher in the older mice (102 total in 30-week-old vs. 18 in 5-week-old mice) (**Figure 3E**). These results provide additional evidence to support the important role of autoimmune reactions in generating 6.9HIP in β-cell granules.

### ER stress promotes 6.9HIP generation

The diabetic autoimmune process is strongly associated with ER stress in β-cells (27–31). The observation of enhanced 6.9HIP presence in inflamed islets led us to examine the impact of ER stress signals on 6.9HIP formation in crinosomes and DCGs. We employed an antigen presentation system designed to quantitatively measure the level of 6.9HIPs in crinosome and DCG subcellular fractions purified from primary islets.

**Figure 4:**
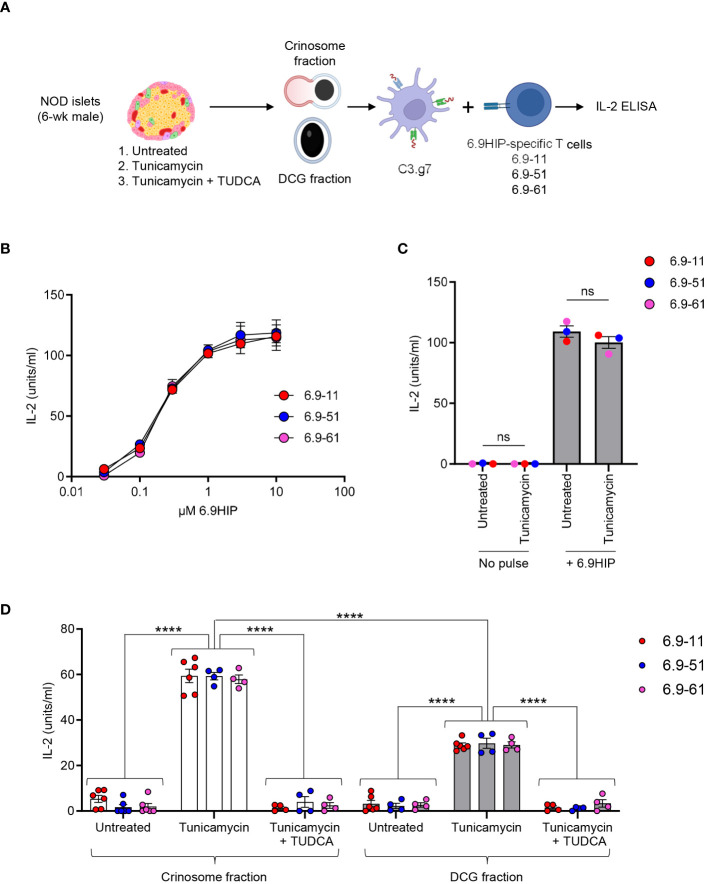
ER stress promotes the production of 6.9HIP in β-cell granules. **(A)** Schematic for assessing the level of 6.9HIP in crinosome and DCG fractions isolated from islets of 6-week-old male NOD mice in the presence or absence of tunicamycin and TUDCA. **(B)** Responses of three 6.9HIP-reactive CD4 T-cell hybridomas to C3.g7 APCs pulsed with indicated concentrations of the 6.9HIP peptide. **(C)** Responses of three 6.9HIP-reactive T-cell hybridomas that were untreated or exposed to tunicamycin for 2 h, in the absence or presence of exogenous pulse with the 6.9HIP peptide (10 µM). **(D)** Responses of the three 6.9HIP-reactive T cells to either the crinosome or the DCG subcellular fractions isolated from islets that were untreated, treated with tunicamycin alone, or treated with both tunicamycin and TUDCA. Data summarize results from three independent experiments; each point represents a biological replicate of a granule fraction. ns, not significant; ****p < 0.001; two-way ANOVA with Sidak’s multiple comparisons test.

Specifically, we prepared dispersed islet cells from 4- to 6-week-old male NOD mice, followed by equilibration in media containing the physiological concentration of glucose (5.5 mM) for 16 h. The islet cells were then left untreated or briefly exposed to the ER stress inducer, tunicamycin (1 µg/mL), for 2 h, a treatment protocol that does not induce β-cell death (data not shown). To confirm the specificity of ER stress, we pretreated one aliquot of islet cells with TUDCA, a well-established ER stress inhibitor during the equilibration period before tunicamycin exposure. TUDCA has been demonstrated to attenuate ER stress and inhibit diabetes development in NOD mice (29). Islet cells under the three conditions were collected, washed, and then subjected to differential centrifugation to isolate subcellular fractions containing crinosomes or DCGs, using our previously established protocol (8). The two fractions were then offered to the C3.g7 APC line (B-cell lymphoma expressing I-Ag7) for antigen presentation to 6.9HIP-reactive, I-Ag7-restricted CD4 T-cell hybridomas. After 24 h, ELISA was used to determine culture IL-2 concentrations for a relative measure of 6.9HIP presence under each condition (see **Figure 4A** for details).

For this study, we generated three 6.9HIP-specific CD4 T-cell hybridomas (6.9-11, 6.9-51, and 6.9-61), each showing dose-responsive reactivity to C3.g7 pulsed with serially diluted concentrations of the synthetic 6.9HIP peptide *in vitro* (**Figure 4B**). In the absence of peptide pulse, treatment of each T-cell hybridoma with tunicamycin did not induce any response (**Figure 4C**). In contrast, exogenous pulsing with 6.9HIP elicited strong and similar responses from all three hybridomas, regardless of tunicamycin exposure (**Figure 4C**). Thus, the T-cell hybridomas were specific for the 6.9HIP peptide and did not react non-specifically to tunicamycin.

All three 6.9HIP-specific T-cell hybridomas responded at background levels to either the crinosome or the DCG fraction from untreated islets from young male NOD mice (**Figure 4D**), confirming that 6.9HIP production is minimal when islet inflammation is low. In contrast, all 3 hybridomas exhibited significantly stronger responses to either crinosomes or DCGs from tunicamycin-treated islet cells (**Figure 4D**). Of note, although crinosomes are anticipated to be much lower in number than DCGs, the level of the T-cell response triggered by the tunicamycin treatment was significantly higher in the crinosome compared to the DCG fraction (**Figure 4D**). Furthermore, TUDCA completely blocked the tunicamycin-induced increase in the presentation of the 6.9HIP in both crinosomes and DCGs (**Figure 4D**), validating the specificity of the observed effects by tunicamycin. Collectively, these data demonstrated that ER stress promoted the concentration of 6.9HIP in both crinosomes and DCGs from islets of NOD mice, with a more pronounced effect observed in crinosomes.

## Discussion

There is increasing evidence that HIPs may be important antigenic peptides in type 1 diabetes. There have been multiple reports of their role in the NOD mouse, demonstrating that HIPs are present in β-cells and are presented to T cells, and that HIP-reactive T cells are key players in disease (11–14, 16, 26, 32). Other studies have shown that HIPs, when presented in tolerogenic fashion, can prevent the adoptive transfer of diabetogenic T cells and protect against destruction of islet isografts in diabetic NOD mice (33, 34). HIPs have also been detected in human islets and HIP-specific T cells with inflammatory phenotypes have been isolated from the PBMCs of T1D patients (12, 35). T cells reactive to HIPs may also serve as important biomarkers of disease as they can be detected in PBMCs taken from subjects at risk for developing T1D (R. Baker et al., unpublished). The accumulating information on HIPs as autoantigens in diabetes thus points to the importance of understanding how these peptides contribute to disease.

In this report, we have described the localization of a HIP within specific islet β-cell subcellular compartments, particularly the DCGs and crinosomes. Although staining with the antibody to insulin B9-23 showed that this peptide could be detected in nearly all of the islets, evidence for the presence of the 6.9HIP was obvious only in the infiltrated islets and in later stages of disease. Micrographs of islets stained with the 6.9HIP MAb, together with antibodies to detect insulin, macrophages, and dendritic cells, indicate that 6.9HIP co-localizes in intra-islet APCs as well as within insulin-positive β-cells. By EM, the 6.9HIP was seen to co-localize with granule structures containing insulin alone or both insulin and LAMP1 within β-cells. Through antigen presentation assays, we could demonstrate that exposure of NOD islets to the ER stress inducer tunicamycin significantly increased the levels of 6.9HIP in subcellular fractions containing DCGs and crinosomes.

These findings are significant for a number of reasons. Firstly, where HIPs are located in the islets has been a looming question since the discovery of these peptides was first reported (13). Although previous biochemical and mass spectrometric analyses have all pointed to the β-cell granules as the origin of HIPs, there has also been evidence of HIPs in APCs and, as shown here, in crinosomes. It is likely that HIPs are produced in the β-cell granules as the pH and densely packed protein environment, as well as the presence of key proteases necessary for formation of HIPs, are all conducive to their formation. Although it is not known exactly how HIPs get taken up into other cellular bodies, the multiple stresses in β-cells incurred during disease-induced inflammation could provide various pathways for these peptides to get processed from granules into other organelles, leading to apoptosis and presentation as antigen by intra-islet APCs. Our results showing that inducing stress in NOD islets leads to increased 6.9HIP levels is in line with the last point.

Here, we used the 6.9HIP MAb to analyze the localization of the 6.9HIP by confocal microscopy and immunogold EM. A key challenge in such studies is the generation of peptide-specific MAbs with satisfactory specificity and applicability. While our study is limited to analysis of a single HIP sequence, such information could potentially be extended to other HIPs, such as the 2.5HIP recognized by the highly pathogenic BDC-2.5 CD4 T-cell clone. Because tetramer-binding CD4 T cells reactive to either 2.5HIP or 6.9HIP were shown to accumulate in the islets as diabetes advances (26), the production of the 2.5HIP may also be driven by islet inflammation, as observed by the increased levels of 6.9HIP in stressed islets in the current study. It is, however, important to acknowledge that different HIPs may exhibit variations in their subcellular locations, modes of presentation, and dynamics of T-cell recognition. Despite these potential differences, disease-relevant HIPs like the 2.5HIP and 6.9HIP are likely to share key characteristics in their involvement in the autoimmune processes of T1D. Understanding these commonalities and differences may be crucial for elucidating the mechanisms of disease progression in T1D and developing targeted therapies.

The discovery that 6.9HIP is predominantly localized within DCGs and crinosomes in β-cells provides new insights into our understanding of T1D pathogenesis. This specific localization suggests that the mechanisms governing the release and processing of HIPs might be intricately linked to those controlling insulin secretion and processing. This connection also sheds light on how HIPs, despite their low abundance, become accessible to the immune system. Our previous studies have demonstrated that islet-resident macrophages are capable of taking up intact secretory granules from β-cells (36), highlighting their prominent role in presenting insulin-derived epitopes in pancreatic islets (36–38). This finding offers intriguing possibilities about the pathways through which HIPs are acquired and presented by intra-islet APCs. One potential mechanism is through direct uptake of granules by islet macrophages possibly during phagocytosis of dying β-cells. Such a process could promote shuttling of granules directly into endocytic compartments for processing and presentation by intra-islet APCs. Alternatively, HIPs might be exocytosed along with insulin peptides as β-cells degranulate when responding to glucose stimulation (8, 19, 39). This exocytosis process could provide intra-islet APCs with access to extracellular HIPs for direct peptide loading onto MHC-II molecules either at the cell surface or within the recycling endosomes, as previously seen with degraded insulin peptides (8, 40–43). This pathway suggests a more dynamic interplay between β-cells and APCs in the islet, where the secretion of β-cell granules directly influences the antigenic landscape encountered by the immune system. In summary, our data suggest that the localization of HIPs in β-cell granules may provide a guided mode of presentation to facilitate efficient recognition of HIPs by the immune system as low-abundance antigens.

## Data availability statement

The raw data supporting the conclusions of this article will be made available by the authors, without undue reservation.

## Ethics statement

The animal study was approved by Division of Comparative Medicine at Washington University School of Medicine Association for Assessment and Accreditation of Laboratory Animal Care accreditation no. A3381-01. The study was conducted in accordance with the local legislation and institutional requirements.

## Author contributions

JW: Conceptualization, Investigation, Resources, Supervision, Validation, Writing – original draft, Writing – review & editing. OP: Investigation, Methodology, Validation, Writing – review & editing. AV: Investigation, Methodology, Validation, Writing – review & editing. JD: Investigation, Methodology, Writing – review & editing. AH: Investigation, Methodology, Validation, Writing – review & editing. KH: Funding acquisition, Project administration, Resources, Supervision, Writing – original draft, Writing – review & editing. XW: Conceptualization, Funding acquisition, Project administration, Resources, Supervision, Writing – original draft, Writing – review & editing.

## Supplementary material

Supplementary Figure 1 Validation of the 6.9HIP MAb in NOD.*Rag1*^-/-^ mice. Intact islets from NOD.*Rag1*^-/-^ mice (27-week-old) were stained for 6.9HIP **(A)** or B9-23 **(B)** along with the macrophage marker F4/80. The islets were then visualized by confocal microscopy. Data are representative of two independent experiments using 4 mice. White scale bars are 50 µM.

Supplementary Figure 2 Validation of primary antibodies for immunogold electron microscopy. NOD mouse (30-week-old) islets were single-labeled with the primary mouse anti-6.9HIP **(A)**, rat anti-LAMP1 **(B)**, or rabbit anti-insulin **(C)** antibody, followed by addition of all three secondary antibodies conjugated to colloidal gold, including anti-rabbit (6-nm), anti-rat (12-nm), and anti-mouse (18-nm). Each enlarged image shows a granule that contains 6.9HIP (18-nm; **A**), LAMP1 (12-nm; **B**), or insulin (6-nm; **C**).
