# Supplementary figures and images for: Mapping of a hybrid insulin peptide in the inflamed islet β-cells from NOD mice
Source: Front Immunol. 2024 Feb 22;15:1348131. doi: 10.3389/fimmu.2024.1348131 (PMC10917911; doi:10.3389/fimmu.2024.1348131)

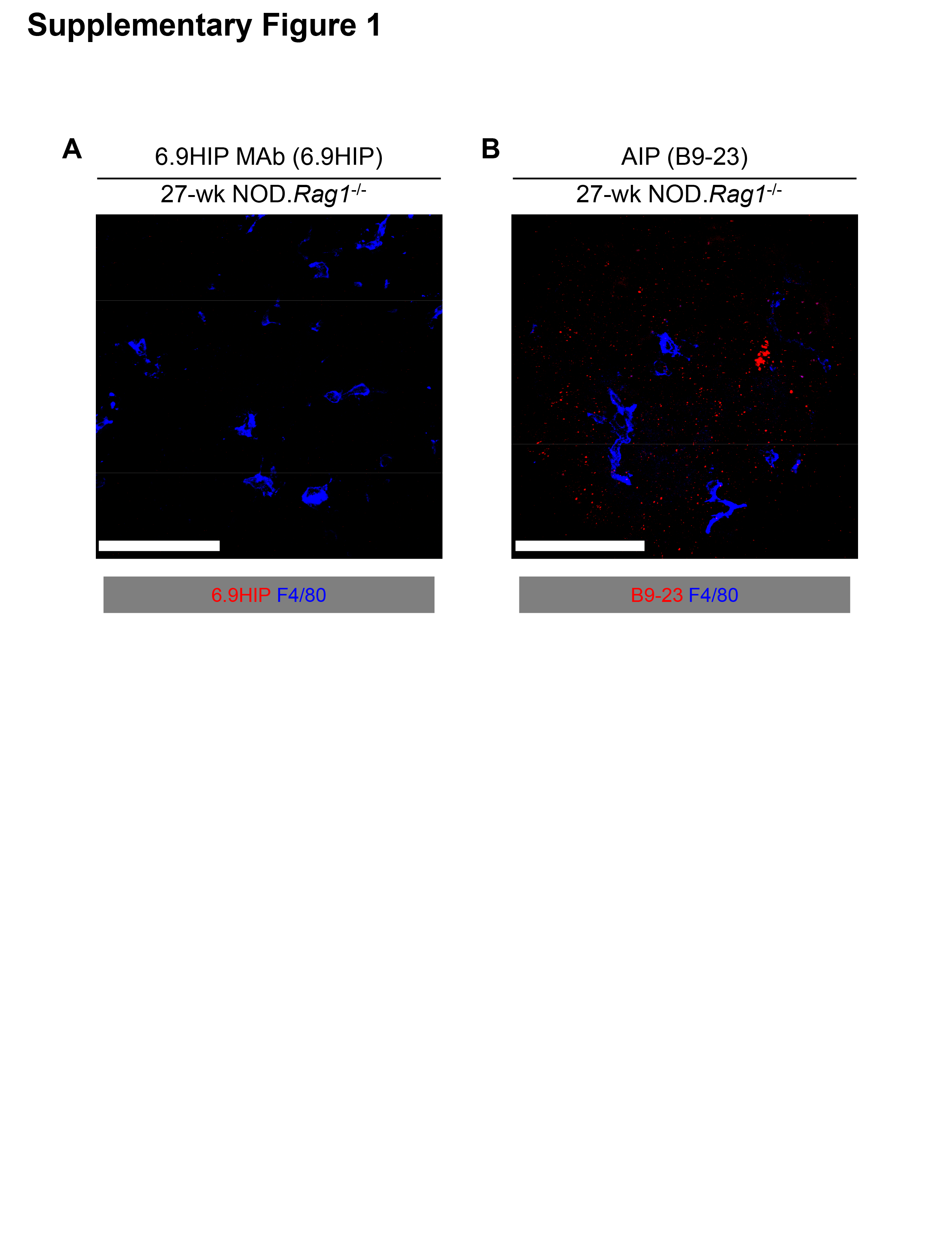

Supplement: Supplementary Figure 1 — Validation of the 6.9HIP MAb in NOD.Rag1-/- mice. Intact islets from NOD.Rag1-/- mice (27-week-old) were stained for 6.9HIP (A) or B9-23 (B) along with the macrophage marker F4/80. The islets were then visualized by confocal microscopy. Data are representative of two independent experiments using 4 mice. White scale bars are 50 µM. [file Image_1.tif]

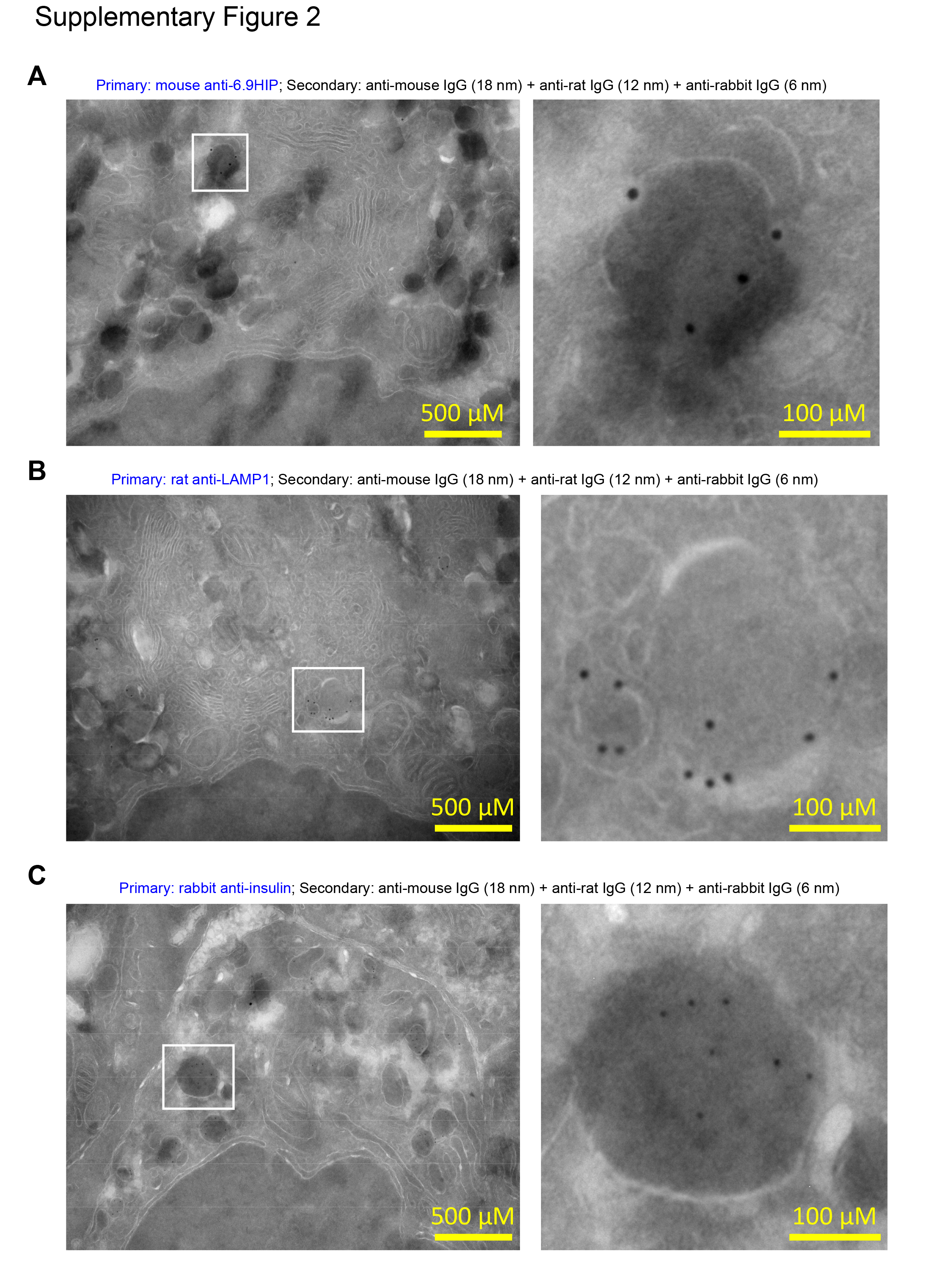

Supplement: Supplementary Figure 2 — Validation of primary antibodies for immunogold electron microscopy. NOD mouse (30-week-old) islets were single-labeled with the primary mouse anti-6.9HIP (A), rat anti-LAMP1 (B), or rabbit anti-insulin (C) antibody, followed by addition of all three secondary antibodies conjugated to colloidal gold, including anti-rabbit (6-nm), anti-rat (12-nm), and anti-mouse (18-nm). Each enlarged image shows a granule that contains 6.9HIP (18-nm; A), LAMP1 (12-nm; B), or insulin (6-nm; C). [file Image_2.tif]
